# Vulnerability of the Hippocampus to Insults: Links to Blood–Brain Barrier Dysfunction

**DOI:** 10.3390/ijms25041991

**Published:** 2024-02-06

**Authors:** Terry L. Davidson, Richard J. Stevenson

**Affiliations:** 1 Department of Neuroscience, Center for Neuroscience and Behavior, American University, 4400 Massachusetts Avenue, NW, Washington, DC 20016, USA; 2 Department of Psychology, Macquarie University, Sydney, NSW 2109, Australia; dick.stevenson@mq.edu.au

**Keywords:** memory, neurodegeneration, neurovascular unit, pathophysiology

## Abstract

The hippocampus is a critical brain substrate for learning and memory; events that harm the hippocampus can seriously impair mental and behavioral functioning. Hippocampal pathophysiologies have been identified as potential causes and effects of a remarkably diverse array of medical diseases, psychological disorders, and environmental sources of damage. It may be that the hippocampus is more vulnerable than other brain areas to insults that are related to these conditions. One purpose of this review is to assess the vulnerability of the hippocampus to the most prevalent types of insults in multiple biomedical domains (i.e., neuroactive pathogens, neurotoxins, neurological conditions, trauma, aging, neurodegenerative disease, acquired brain injury, mental health conditions, endocrine disorders, developmental disabilities, nutrition) and to evaluate whether these insults affect the hippocampus first and more prominently compared to other brain loci. A second purpose is to consider the role of hippocampal blood–brain barrier (BBB) breakdown in either causing or worsening the harmful effects of each insult. Recent research suggests that the hippocampal BBB is more fragile compared to other brain areas and may also be more prone to the disruption of the transport mechanisms that act to maintain the internal milieu. Moreover, a compromised BBB could be a factor that is common to many different types of insults. Our analysis indicates that the hippocampus is more vulnerable to insults compared to other parts of the brain, and that developing interventions that protect the hippocampal BBB may help to prevent or ameliorate the harmful effects of many insults on memory and cognition.

## 1. Vulnerability of the Hippocampus to Insults: Links to Blood–Brain Barrier Dysfunction

The hippocampus, a medial temporal lobe structure that is a critical substrate (i.e., central nervous system component) that underlies learning and memory functions, can be adversely affected by a wide range of pathogens, neurotoxins, diseases, injuries, and environmental insults. It has often been suggested that the harmful effects of these insults may be greater on the hippocampus compared to other brain areas [1,2,3,4,5]. However, there has been no systematic examination of this claim. An important reason to conduct this examination is that Alzheimer’s disease and the severe dementia it causes are characterized by extensive hippocampal pathophysiology. It may be that insults that impair hippocampal functioning earlier in life may accelerate the emergence of more extensive hippocampal pathologies that could increase the risk of serious late-life cognitive decline. The purpose of the present study is to identify the types of insults to which the hippocampus is vulnerable, to assess the primacy and the pervasiveness of their detrimental effects on the hippocampus, relative to other brain structures, and to explore the role of blood–brain barrier disruption in the manifestation of those undesirable consequences.

To systematically address whether the hippocampus may be more vulnerable to insult than other brain areas requires that we know, for any given agent or event, at least three things. First, we must know what other brain structures are affected. Second, we need to determine whether the hippocampus is the most impacted, relative to other structures. Third, we need to determine whether the hippocampus was damaged first, even if it is not more damaged than the other structures. Furthermore, although many insults have interrelated pathophysiological consequences—such as inflammation, oxidative stress, and excitotoxicity [6], which can be potent sources of neurodegeneration [7,8]—the matter of why the hippocampus is more susceptible to these consequences compared to other brain regions also needs to be considered. 

To begin our analysis, we identified all the major biomedical domains that are known to affect brain function (see Table 1). We then selected two (or more if necessary) examples of insults from each domain for study. As a guard against bias, the examples we selected were the most prevalent (i.e., affected the most people) in each domain. In addition, for each of the examples chosen, we reviewed the evidence pertaining to whether their harmful effects caused, were caused by, or were exacerbated by, a compromised hippocampal BBB. As the literature is substantial (e.g., in PubMed, the search term “hippocampus” returned 186,446 results and the term “blood–brain barrier” returned 63,913), a formal PRISMA guidelines systematic review for each domain and its examples would be unfeasible. Accordingly, for each example, our search strategy was to first identify all formal systematic and narrative reviews, and/or meta-analyses, which examined the following question: how does the insult affect the brain? Second, via these reviews/meta-analyses, we examined key cited papers that contrasted multiple brain areas and/or studied the order in which particular brain structures were damaged. Third, in the absence of overarching reviews, and in addition to the first two steps, we searched for studies that had the insult in the title with the terms “brain” and “hippocampus”. To evaluate whether the integrity of the BBB was compromised for each example, in the hippocampus selectively, or as part of a more global disruption, we used the PubMed database to search for publications that included the name of the example combined with the terms “blood–brain barrier” and “hippocampus” in the title or abstract.

### Why Focus on the Hippocampal BBB?

The BBB is comprised of endothelial cells that are supported by and interact with tight-junction proteins, pericytes, astrocytes, and microglia, to form a physical low-permeability boundary that protects and maintains the internal milieu of the brain [9,10]. Collectively referred to as the neurovascular unit (NVU), dysfunction in any of these components can result in neuronal injury or disease [11]. Of special interest is the fact that the protections afforded by the BBB are not uniform across all brain regions [12]. For example, it has been suggested that, due to the intrinsic fragility of its vascular network, the hippocampal BBB is less able to cope with hypoperfusion and anoxia compared to other cortical areas [13]. Furthermore, a study using dynamic contrast-enhanced MRI (DCE-MRI)—an imaging technique that can measure brain uptake of a tracer or contrast agent which does not cross the BBB under normal physiological conditions—showed that BBB permeability in the hippocampus was higher compared to several other brain regions, even in human subjects with no known brain pathology [14]. Montagne and his collaborators [15,16] also used DCE-MRI to reveal a strong positive correlation between age and BBB permeability in the total hippocampus, the CA1 subfield, and the dentate gyrus of adult humans. While this correlation was also found to be significant for the caudate nucleus, it failed to reach significance in the hippocampal CA3 region, the superior frontal, inferior temporal cortical gyri, striatum, thalamus, subcortical white matter fibers, corpus callosum, and internal capsule. In summary, these findings indicate that the BBB may provide less protection to the hippocampus and hippocampal formation relative to other brain structures. Importantly, this suggests that BBB weakness may underlie the potential selective vulnerability of the hippocampus to diverse types of insults. Moreover, the BBB regulates the transport into the brain of glucose and hormones (e.g., insulin, ghrelin, leptin) that serve to maintain hippocampal function [17]. There is evidence that interference in these BBB transport mechanisms may contribute to the deleterious effects of insults on hippocampal-dependent memory [18].

## 2. Vulnerabilities of the Hippocampus

### 2.1. Neuroactive Pathogens

The two most important neuroactive pathogens are rabies and herpes simplex virus (HSV I and II). Rabies kills 20,000–70,000 people/year in the developing world [19,20], making it the most lethal neuroactive pathogen. In the developed world, the most significant neuroactive pathogen is HSV, the commonest cause of sporadic encephalitis [21,22].

#### 2.1.1. Rabies

Rabies migrates along peripheral neurons to the spinal cord and then to the brain [20]. The brain stem is affected initially [23,24], followed by several other areas as the disease progresses, notably the hippocampus, hypothalamus, and white matter tracts [24,25,26]. While there is unambiguous evidence of hippocampal damage, a conclusion supported by animal model data is that the hippocampus is neither the first nor most damaged site. 

Rabies and the BBB: There appears to be only one study that has examined the effects of rabies on the BBB. Using MRI, Laothamatas et al. [27] reported that BBB leakage is observed as soon as rabies-virus-infected humans and dogs became comatose, but not when they remained conscious. However, to date there have been no reports that identifies rabies with the breakdown of the hippocampal BBB.

#### 2.1.2. Herpes Simplex Virus (HSV)

HSV I and II is prevalent in humans and generally without major health impacts [28]. However, in some cases, HSV leads to the development of herpes simplex encephalitis (HSE) [29], with the virus entering the brain via the trigeminal nerve or the olfactory system [30]. HSE is associated with a high mortality rate and serious neurological damage [31]. 

Several sources of evidence indicate that HSV’s initial site of action, and the site it damages most, is the hippocampus. First, animal models indicate that HSV’s primary target are the granule cells of the dentate gyrus [32]. Second, HSV infection may be a risk factor for Alzheimer’s disease [33], with the virus’s propensity for the hippocampus being of special note [34]. Third, in both animals and humans, neuroimaging during active and confirmed HSE indicates abnormalities in the medial temporal lobes [29,35,36,37]. Fourth, residual memory deficits of the sort associated with hippocampal damage have been reported in individuals who recover from HSE [38]. 

HSV and the BBB: The brain damage produced by HSV has been attributed to inflammatory reactions to the virus, to toxic products of viral replication that result in glial and neuronal death, and, more recently, to the degradation of the BBB [39]. Similar to epilepsy and ischemia, degradation of the BBB in patients with HSE and in nonhuman animal models of HSV-1 infection are accompanied by significant upregulation of matrix metalloproteinases (MMPs). These weaken the BBB by cleaving tight-junction proteins [40,41,42], thereby facilitating the migration of both proinflammatory cytokines and the viral agent from the sera to the brain parenchyma [37,43]. Evidence from human cell culture models indicates that HSV also damages the BBB by reducing the viability of microvascular endothelial cells of the infected brain [44]. However, while there is clear evidence that HSV can disrupt the BBB, there appear to be no studies that have investigated the effects of HSV on the hippocampus directly or that compared the effects of HSV on the hippocampus with its effects on other brain areas.

Rabies and HSV—Conclusions: The hippocampus is damaged by rabies, but it is not the initial site of damage, nor does it seem the most prominent. In contrast, the hippocampus is an initial and prominent target of HSV. Considering the BBB, rabies may have an adverse effect on the BBB, at least under limited conditions, but whether rabies has any selective effect of the hippocampal BBB is unknown. In contrast, the available evidence indicates that HSV may have a strong disruptive effect on the BBB. However, as for rabies, direct evidence is lacking on whether the viral agent disrupts the hippocampal BBB either independently or as part of a more global effect on the brain. 

### 2.2. Neurotoxins

We are exposed to many chemicals whose neurotoxicity is largely unknown [45]. This precludes any representative survey. To try and reflect the breadth of agents in this domain, four were selected, based on their current or historical significance. Alcohol (ethanol) was picked due to its widespread use among Western populations [46]. Arsenic was selected because it is a major health risk in many developing countries [47]. For example, in Bangladesh, between 25 and 50% of the population are exposed to arsenic-contaminated water [48]. Pesticides were included as many have both acute and chronic neurotoxic effects, with the most investigated being organophosphate (OP) and organochloride (OC) insecticides [49]. Finally, both organic and inorganic forms of lead (Pb) have long been recognized as neurotoxins [50], hence their inclusion too.

#### 2.2.1. Alcohol

Chronic alcohol use damages frontal areas, the limbic system, including the hippocampus and amygdala, and the cerebellum [51,52]. There have been some suggestions that the prefrontal cortex and the hippocampus may be especially sensitive to the chronic effects of alcohol [53]. Alcohol impairs neurogenesis [54]; it is associated with an accelerated reduction in hippocampal volume in longitudinal studies of people who drank heavily when they were young [55]. Indeed, a recent meta-analytic review indicated that smaller hippocampi were associated with chronic alcohol consumption [56], with this being confirmed in more recent studies [57]. However, there are two reasons for caution. First, alcohol is a ‘promiscuous’ drug, with multiple effects on the brain [58,59]. Second, animal data suggest that chronic use manifests initially in the prefrontal cortex [60]. To summarize, the hippocampus is clearly impacted by alcohol use, but not first or uniquely so.

Alcohol consumption and BBB: With respect to BBB function, one mouse study reported that 30-day alcohol consumption increased BBB permeability and decreased the expression of tight-junction proteins [61]. Another study found that wild-type mice exposed to a binge-like alcohol consumption protocol exhibited increased hippocampal BBB permeability, which appeared to be mediated by Toll-like receptor 4 neuroinflammatory signaling [62]. However, it was also found that a four-day alcohol binge produced no evidence of heightened BBB permeability or inflammation in the rat hippocampus or entorhinal cortex [63]. A more recent finding with rats is that intragastric administration of alcohol increased BBB permeability in the hippocampus, but also in the nucleus accumbens, cingulate prefrontal cortex, amygdala, and the ventral tegmental area [64]. In summary, there is some evidence that excessive alcohol intake disrupts BBB functioning, but evidence is lacking that this effect is selective for the hippocampal BBB. 

#### 2.2.2. Arsenic

Most human exposure to arsenic is chronic, and to its inorganic form [65]. In children, arsenic exposure is associated with detrimental effects to IQ, and with reduced performance across all cognitive domains, including hippocampal-dependent learning and memory (HDLM) [66]. In adults, epidemiological studies, notably the FRONTIER study in West Texas, have found general impairments in cognition, including in tests of HDLM [67,68,69]. These studies are not informative as to which aspects of functioning are affected first. One human study does shed some light on this question. Bolla-Wilson and Bleecker [70] report a case of industrial arsenic exposure, with neuropsychological functioning studied over the recovery period. The most significant initial deficit was in HDLM, which recovered little, in contrast to other cognitive domains.

A further means of addressing whether the hippocampus is a primary/initial target of arsenic is to examine the data from nonhuman animals. A systematic review [71] concludes that the hippocampus is a primary target. Arsenic exposure affects the results of multiple behavioral tests that are sensitive to hippocampal function [72] and neurogenesis [73]. There has also been interest in whether arsenic exposure is a risk factor for Alzheimer’s disease [74]. Rodent models suggest that arsenic exposure can be a risk factor [75], as do human data [76], further implicating the hippocampus as a target for arsenic. In summary, the hippocampus is prominently affected by arsenic [70,71], and some data suggest it may be affected first.

Arsenic and the BBB: The results of several studies indicate that arsenic weakens the BBB. For example, BBB permeability was markedly increased in the brains of mice that received arsenic in their drinking water, relative to nontreated controls. Although BBB permeability was measured globally, this study also found that arsenic reduced levels of the tight-junction proteins claudin-5 and occludin in the cortex and hippocampus. However, the reduction in occludin occurred with lower doses of arsenic in the cortex compared to the hippocampus [77]. In another study, while BBB permeability was not measured, reduced expression of occludin in the hippocampal BBB was observed in mice that received arsenic intragastrically. This effect was accompanied by elevated levels of proinflammatory cytokines also in the hippocampus [78]. In addition, in a developmental study, arsenic in the drinking water (0.15, 1.5, and 15 mg) reduced levels of occludin and claudin-5, along with two other tight-junction proteins, ZO-1 and Z0-2, in the hippocampus and cortex of mice at postnatal days 21, 28, 35, and 42. The largest reductions were observed at postnatal day 21 [79]. Furthermore, arsenic produced evidence of tissue injury in both the hippocampus and the cortex at the highest dose tested. Considered together, these data indicate that arsenic weakens the BBB in the hippocampus, and this effect may be more pronounced the cortex. 

#### 2.2.3. Organochloride (OC) and Organophosphate (OP) Insecticides

Both acute and chronic exposure to OC/OP insecticides can impair cognitive function, including memory [80,81]. In a large study, farm workers who reported an acute poisoning episode showed impairment relative to controls on the Benton visual retention test, tests of executive function, and tests of fine motor performance [82]. The Benton test is considered an index of hippocampal-dependent learning (HDLM) [83]. A systematic review/meta-analysis of the chronic effects of OC/OP exposure on cognition examined 22 mainly cross-sectional studies [84]. The largest effect size was for the Benton visual retention test, followed by tests of executive function and fine motor performance. Three conclusions emerged. First, around half the studies found deficits in spatial memory tasks (i.e., HDLM). Second, the most consistent deficits were found to be for fine motor skills and balance. Third, hippocampal impairments were found to be persistent [85]. This persistence may be important in the light of OC/OP exposure being linked to neurodegenerative disease [86,87,88]. Lin et al. [89] followed a large cohort of individuals who had been hospitalized for acute OC/OP poisoning. Relative to controls, they were significantly more likely to develop Alzheimer’s disease during the decade following their hospitalization. In summary, acute and chronic OC/OP exposure damages the hippocampus, but it is not clear whether this occurs first or more so than for other brain areas.

OC/OP insecticides and BBB: Only a few studies have investigated the effects of OC/OP insecticides on BBB integrity. The available experimental data suggest that the effects of OC/OP exposure on BBB permeability may vary based on age of exposure and species. For example, Gupta et al. [90] found that 10-day old rats exposed to OC/OP compounds exhibit increased BBB permeability, whereas adult rats did not [90]. Further, Sinha and Shukla [91] also found OC/OP pesticides did not alter BBB permeability in adult rats but did increase extravasation of sodium fluorescein (NaFl; a small molecule dye) across the BBB in the brains of mice. However, there appear to be no studies that have investigated the effects of OC/OP pesticides specific to the hippocampal BBB.

#### 2.2.4. Lead (Pb)

Four conclusions emerge from the literature on Pb exposure. First, in adults, Pb is associated with multiple cognitive impairments [50,92,93], suggesting its impact is unlikely to be restricted to one area. This view is further supported by neuroimaging [94] and by the literature on childhood Pb exposure [95,96]. Second, impaired HDLM is consistently observed [50,92,93]. Third, animal studies suggest that multiple brain areas are damaged by Pb exposure [97,98], including the hippocampus [99]. However, among studies that contrast multiple brain areas, some find evidence of hippocampal damage occurring selectively [100,101]. Fourth, Pb has also been suggested as a potential environmental cause of Alzheimer’s disease [102,103]. In conclusion, Pb clearly impacts the hippocampus, alongside many other brain areas. There is some limited evidence that it may do so first.

PB and the BBB: Pb easily crosses the BBB and accumulates in brain cells in the hippocampus and elsewhere [104]. BBB damage is also known to be a consequence of Pb toxicity, with Pb-induced barrier loss and decreased expression of tight-junction proteins observed in developing rat brains [105]. Although the hippocampus is a site where inorganic Pb compounds collect relative to other brain areas [106], it is currently unknown whether Pb damages the hippocampal BBB first or most prominently compared to other brain loci. 

Alcohol, Arsenic, OC/OP Pesticides, and Pb—Conclusions: Alcohol, arsenic, OC/OP insecticides, and Pb all result in significant hippocampal impairment—alongside the impairment of other brain areas. The strongest case for hippocampal prominence (i.e., first and most affected) is for arsenic. Similarly, among these four neurotoxins, the best evidence for hippocampal BBB disruption comes from studies evaluating the effects of arsenic. However, even with arsenic, the findings do not indicate that the hippocampal BBB is affected more than the other areas. Interestingly, all these neurotoxins, except for alcohol (which has a separate association with dementia), have been suggested as environmental causes of Alzheimer’s disease; this is notable, because the hippocampus is an early target for this disease (see below). 

### 2.3. Neurological Conditions

The Global Burden of Disease Study 2010 data [107] found that migraine and epilepsy were ranked first and second, respectively, among neurological conditions, in terms of years lived with disability (i.e., prevalence multiplied by a weight representing the degree of imposed disability)—hence their inclusion here.

#### 2.3.1. Epilepsy

Epilepsy, defined as having two or more unprovoked seizures [107,108], can result from multiple environmental and genetic causes [109,110,111]. The most relevant form of seizure classification is between generalized and partial, with the latter referred to as focal epilepsy [109]. As this name implies, focal seizures tend to be associated with damage to a part of the brain in contrast to generalized seizures (not including generalized seizure resulting *secondarily* to partial seizures). The most common site of epileptogenic foci are structures within the medial temporal lobes and especially the hippocampus [112]. 

At least half of people diagnosed with medial temporal lobe epilepsy have identifiable lesions termed hippocampal sclerosis [113,114,115], the most common type of focal damage in epilepsy. As to whether hippocampal sclerosis is a cause or a consequence of focal epilepsy, it appears to be both. Multiple genetic factors predispose people to epilepsy [109], with hippocampal insults increasing the risk of sclerosis, which in turn risks the hippocampus becoming a seizure focus; this results in multiple seizures, further sclerosis, and increased risk of additional seizures [116,117,118]. In sum, the hippocampus is the initial and most damaged site for most focal epilepsies, and this is linked to a greater risk for AD [119].

Epilepsy and the BBB: While it has long been known that seizures can alter the BBB [120], the specific regional loci as well as the time course for BBB damage has received only recent attention. Some studies have measured the extravasation of dyes, such as Evans Blue or NaFl, for 5–30 min following acute, chemically induced seizures. They have observed large increases in BBB permeability in limbic areas of the brain, including the hippocampus, that are the initial sites of epileptic activity [121]. There is also evidence from human and nonhuman animal models that BBB disruption may promote insult-induced epileptogenesis. For example, osmotic disruption of the BBB is used to increase the penetration of chemotherapeutic agents in patients with brain lymphomas. Marchi et al. [122] reported that seizures are an almost immediate consequence for ~25% of patients that receive this treatment. The same paper provided additional, compelling evidence that seizure activity is a result of BBB opening by using a porcine model to show that osmotic disruption of the BBB was accompanied by the rapid onset of seizures when both chemotherapeutic drugs and brain tumors were excluded. Other studies find that, while increased BBB permeability occurs within minutes following experimentally induced status epilepticus (SE), spontaneous seizures are not observed until much later [123]. These findings support the conclusion that, like hippocampal sclerosis, BBB disruption can be both a consequence and a cause of epileptic activity [124]. 

Although epilepsy is associated with damage that occurs first and most prominently in the hippocampus, there is evidence that the hippocampal BBB may not be the first and foremost site of seizure-induced BBB disruption. In some SE models, the hippocampus, amygdala, and piriform and entorhinal cortices are viewed as the main components of the limbic circuit or network that drives seizure activity [125]. As assessed by magnetic resonance imaging, BBB leakage in an experimental model of temporal lobe epilepsy has been observed in each of these areas, with less leakage detected in the hippocampus compared to the each of the other areas. Moreover, this pattern of differences was observed one day (the acute phase) and, although less pronounced, six weeks (chronic phase) after seizure induction. While seizures affected the whole brain, BBB leakage above control levels was seen only in the limbic areas [123,126]. Similarly, Bernardino [127] reported that BBB permeability in rats was increased at six hours and persisted up to seven days following organophosphate-induced SE. The largest of these effects were observed in the piriform cortex and amygdala and were less severe and more transient in the hippocampus. 

#### 2.3.2. Migraine

Migraine involves an intense unilateral headache, accompanied by increased sensitivity to sensory stimuli and nausea. Four main forms of evidence are pertinent to evaluating the role of the hippocampus in this disorder. The first concerns neuroimaging to identify brain structures that may be involved in migraine [128], which mainly find evidence of hypothalamic [129,130] and brain stem involvement [131]. Only a few report hippocampal involvement [129,132]. A second form of evidence comes from neuropsychological studies. These studies find some executively related deficits [133], and some for HDLM [134]. A third form of evidence concerns genetics, with gene variants that predispose to this disorder overwhelmingly relating to the vascular system [135]. The fourth form of evidence concerns the overlap between epilepsy and migraine, and migraine and transient global amnesia, and that migraine and epilepsy can be controlled with some of the same medications [136]. While this might lead one to suspect greater hippocampal involvement, the imaging, neuropsychological, and genetic evidence suggests otherwise.

Migraine and the BBB: The hypothesis that BBB dysfunction is an important contributor to migraine [137] has generated much research and many conflicting findings [138]. Early neuroimaging studies provided evidence of BBB opening in patients that experience severe migraine headaches preceded by aura [139,140]. This was associated with large-scale reduction in electrical activity that spreads across the gray matter of the brain (i.e., spreading depolarization). Extending these findings, Gursoy-Ozdemir [141] surgically induced spreading depolarization in rodents and opening of the BBB. However, more recent neuroimaging studies using dynamic contrast enhanced (DCE) MRI are less supportive. They find no evidence of BBB leakage in spontaneous migraine with or without aura [142,143] or when comparing migraine patients with healthy controls where scanning took place outside of attacks [144]. Further, in the only study that examined the hippocampal BBB specifically, no changes in permeability were observed in migraine patients during a chemically induced migraine state [144]. In addition, while some studies have reported elevated MMP levels in migraine patients [145,146], other studies have failed to confirm this relationship [147,148]. Overall, inconsistencies in the available data prevent any strong conclusions about the role of BBB dysfunction in migraine, and the only study that examined the effects of migraine on the hippocampus found no evidence of BBB disruption.

Epilepsy and Migraine—Conclusions: Focal epilepsy commonly involves the hippocampus as the first and main site of damage. Epilepsy is also associated with BBB breakdown in the hippocampus, but there is evidence that this breakdown is larger in other parts of the limbic system. For migraine, while some reports indicate that BBB disruption is associated with this disorder, other studies have not, and there appears to be no evidence that links the migraine to changes in the hippocampal BBB. 

### 2.4. Trauma

Early-life stress (ELS) includes child maltreatment and the consequences of exposure to serious adverse events [149]. ELS contributes to the occurrence of a third of all mental disorders [150] and two thirds of all suicides [151], making it the most significant modifiable risk factor for psychiatric illness. Post-traumatic stress disorder (PTSD) is one of the most common anxiety disorders, with a lifetime prevalence in the general US population of around 6%; it also occurs in up to 29% of US veterans (United States Department of Veterans Affairs, 2023). PTSD has strong associations with substance abuse, depression, and increased risk for cardiovascular disease and obesity [152].

#### 2.4.1. Early-Life Stress (ELS)

Three lines of evidence suggest hippocampal damage is an initial and major consequence of ELS [153]. The first is from children who have experienced ELS and who evidence no other concurrent psychiatric illness. They exhibit neuropsychological deficits primarily in learning and memory [154]. For neuroimaging data, a meta-analysis examining just children who had experienced ELS without other current psychopathology found abnormal activation in just the hippocampus and amygdala [155]. A second line of evidence comes from imaging studies involving adults who experienced ELS. A meta-analysis of such studies [156] examined those who were currently healthy (using region-of-interest analysis), finding significant reductions in hippocampal volume in the currently healthy group. However, a far broader meta-analysis of functional imaging studies of survivors of early-life adversity (including pre-natal forms, pre-term birth, and adverse childhood experiences) found no hippocampal effects, and differences were found only in the frontal regions among currently healthy participants [157]. Functional hippocampal abnormalities may not then be evident for all forms of early-life adversity. A third line of evidence comes from animal data, where there have been both systematic reviews [158] and meta-analyses [159] of the extensive literature. Both conclude that the hippocampus is an initial and primary target for ELS, and the human ELS data are largely consistent with these findings. 

ELS and the BBB: The few studies that have investigated the potential links between ELS and alterations in the hippocampal BBB have found inconsistent or, at best, weak relationships. In what appears to be the first study of this type, Gómez-González and Escobar [160] exposed rat pups to ELS through maternal separation (MS) for 180 min daily from postnatal day (PND) 2 through PND 10 or PND 20, after which the rats were sacrificed, and BBB permeability was evaluated. Relative to non-stressed controls, in 10-day old rats heightened BBB permeability, assessed via penetration of Evans blue dye into the parenchyma, was widespread, increasing significantly in the hippocampus and in the thalamus, hypothalamus, basal ganglia, olfactory bulb, midbrain, pons, medulla, and cerebellum. In contrast, at 20 days of age, there were no differences in permeability for any of these regions. Thus, these findings suggest that the effects of ELS on BBB integrity are both transient and not selective for the hippocampus. In a more recent study, using the maternal separation protocol during days 1–14, Solarz et al. [161] compared BBB permeability and the expression of tight-junction proteins in the hippocampus, striatum, and medial prefrontal cortex (mPFC) of juvenile (15-day old) and adult (70-day old) rats. Their overall conclusion—unlike Gómez-González and Escobar who found widespread BBB disruption—was that ELS had no clear or strong negative impact on the integrity of the BBB at either age. A subsequent experiment assessed the effects on BBB function when ELS was combined with a neuroinflammatory challenge [162]. Here, rats exposed to maternal separation during postnatal days 1–14 were administered lipopolysaccharide (LPS) at either 22 or 70 days of age to induce inflammation. BBB permeability and tight-junction protein expression in the mPFC and hippocampus were evaluated 24 h after LPS injection. Maternal separation plus LPS increased BBB permeability in both brain regions of the juvenile rats compared to the controls, who did not experience maternal separation. This effect was accompanied by enhanced expression of claudin-5 and occludin only in the mPFC and only in males. In adult rats, although LPS and maternal separation increased BBB permeability and the expression of tight-junction proteins in the hippocampus relative to controls reared under normal conditions, the effect on BBB permeability was small and was attributable to lower baseline permeability for rats that experienced maternal separation without LPS. These results suggest that, while ELS may combine with environmental insults to impair BBB function early in brain development, that effect is short-lived and not specific to the hippocampal BBB. In summary, there is no basis to conclude that ELS disturbs the hippocampal BBB selectively relative to other brain structures.

#### 2.4.2. Post-Traumatic Stress Disorder (PTSD)

PTSD is associated both with impaired performance on tests of HDLM [163] and by a reduction in size of both hippocampi [164,165]. This latter finding has been established using structural MRI, and while not all individual reports have been consistent, two observations suggest convincingly that hippocampal volume reduction is a correlate of PTSD. First, a large study comparing 794 PTSD patients with 1094 controls, with all data collected according to a standardized protocol, reported significant hippocampal volume reductions in PTSD patients, but no difference in other subcortical structures [166]. Second, a meta-analysis of 39 studies also reported significant hippocampal volume reduction in PTSD patient samples [167]. Whether a smaller hippocampus is a risk factor for or a consequence of PTSD has been contentious [168]. However, several findings [167,169], including from studies of identical twins [170], suggest that environmental factors are more important, indicating that PTSD causes reduced hippocampal volume. It is also noteworthy that there is good evidence for a link between AD and PTSD [171]. Animal data are also consistent with this, as acute exposure to life-threatening stressors can result in rapid hippocampal impairment [172]. While the involvement of the hippocampus in PTSD is well established, it is important to note that human and animal research has identified the involvement of other limbic and frontal structures [165,173,174,175,176,177]. Nonetheless, the hippocampus is probably both the initial and most prominently affected structure in PTSD.

PTSD and the BBB: Nonhuman animal models provide data that link PTSD to hippocampal BBB disruption. Rats exposed to a single prolonged stress (SPS) procedure in which they were immobilized inside a plastic container for 2 h followed by a 20 min swim exhibited significant deficits in hippocampal-dependent working memory, relative to unstressed controls. These deficits were accompanied by increased hippocampal BBB permeability and significantly reduced expression of claudin-5 and occludin [178]. In a second experiment in the same study, pretreatment with the antibiotic minocycline protected rats from the detrimental effects of SPS on working memory, blood–brain barrier permeability, and the expression of tight-junction proteins. This finding suggests that the harmful effects of SPS on the BBB and hippocampal-dependent cognition are mediated by microglia activation. Another study [179] found rats that were especially sensitive to stress produced by the SPS procedure also exhibited a significant reduction in claudin 5, but not occludin.

ELS and PTSD—Conclusions: The hippocampus is the initial site of damage, and the most prominently damaged structure, for both ELS and PTSD. Research investigating the effects of ELS on the BBB has produced inconsistent results and studies on the selective effects of ELS on the hippocampal BBB are lacking. The evidence, which has been obtained only from animal models, indicates that disruption of the hippocampal BBB could underlie some of the effects of PTSD.

### 2.5. Aging

Populations around the world are rapidly aging [180] and changes in the aging brain are well established [181], making it important to consider the effects of aging on the hippocampus. Age-related cognitive decline has been documented in many domains [181,182]. However, deficits in episodic memory may be both the first and most prominent signs of cognitive decline with age [181,183]. The hippocampus is considered an important substrate for episodic memory retrieval [184]), and there is substantial evidence that hippocampal-dependent episodic memory functioning deteriorates with age. Indeed, it has been suggested that age-related change in hippocampal function can be used as a criterion for measuring successful aging [183]. For example, cortical grey matter volume decreases as a largely linear function of increasing age [185,186], with frontal, temporal, and medial temporal areas exhibiting, proportionally, the largest reductions [187]. In contrast, the decline in hippocampal volume departs from this linear pattern by markedly accelerating after 60 years of age. This effect seems to be attributable to normal aging and not to neurodegenerative disease [188,189]. Further, meta-analyses reveal the following findings: (a) the relationship between hippocampal volume and episodic memory performance becomes increasingly positive with advancing years [190]; (b) based on functional brain imaging, age-related deficits in episodic memory were most strongly associated with reduced resting state functional connectivity in the default mode network [191], of which the hippocampus is a critical node [192,193]. In summary, these findings suggest that aging may impact the hippocampus first and more prominently compared to other brain areas.

Aging and the BBB: There is also compelling evidence that the BBB weakens with age. Montagne and his collaborators [15,16,194] used DCE-MRI to reveal strong positive correlations between age and BBB permeability in the total hippocampus, the CA1 subfield, and the dentate gyrus of adult humans. While this correlation was also significant for the caudate nucleus, it failed to reach significance in the hippocampal CA3 region, the superior frontal, inferior temporal cortical gyri, striatum, thalamus, subcortical white matter fibers, corpus callosum, and internal capsule. Increased BBB permeability was observed as a function of age in individuals that did not exhibit cognitive impairment. 

Research with rodent models also consistently reveals weakening of the hippocampal BBB with age. For example, Pelegrí et al. [195] found evidence of increased BBB permeability in the hippocampus of 12-month-old mice from a senescence-accelerated mouse strain, compared with controls from a genetically related strain that was resistant to senescence. In another study, measuring the extravasation of a contrast agent, Blau et al. [196] observed an age-related increase in BBB permeability in the hippocampus and not in the cortex of old (age 20–26 months) compared to young (age 3–5 months) Wistar rats. This study also reported that this heightened BBB permeability in the aged mice was associated with reduced hippocampal long-term potentiation (LTP). Because hippocampal LTP is an important part of the cellular machinery underlying learning and memory [197], this association suggests that hippocampal BBB dysfunction could be responsible, in part, for age-related impairments in episodic memory and other hippocampal-dependent cognitive processes. In addition to increased permeability, there is evidence that receptor-mediated transport from plasma across the BBB into the parenchyma is also degraded in the ageing hippocampus [198]. Alterations in the receptor transport of peptides and nutrients into the hippocampal milieu, as well as the transport of waste products out, could also be a basis for age-related deficits in hippocampal functioning. 

A number of studies have investigated the structural changes that underlie the deterioration of the aging hippocampal BBB, with pericyte injury being the focus of much of this research [199]. For example, in the aforementioned study by Montagne et al. [15], humans with MCI and age-matched controls with normal cognitive functioning were compared on CSF levels of soluble platelet-derived growth factor receptor b (sPDGFRb). This is an established biomarker of BBB-associated pericytes [200,201]. Levels of sPDGFRb in the CA1 and dentate gyrus were significantly higher for the MCI patients and were directly correlated with BBB permeability (measured by DCE-MRI) in both subregions. Another study involving members of the same research group [202] reported that a transgenic mouse model exhibited a progressive, age-related loss of pericyte coverage and BBB breakdown, which was not confined to the hippocampus, but was also observed in the cortex and thalamus. Pericycte loss and BBB permeability also increased in the dentate gyrus of mice at 6 and 12 months compared to 2 months of age, suggesting that these changes may contribute to the reduced neurogenesis seen in this region with advancing age [200]. 

There is also evidence that age-related structural changes in hippocampal BBB integrity may be sex-dependent. For example, Bake et al. [203] found evidence of greater BBB permeability in the hippocampus and thalamus of reproductive senescent, middle-aged female and age-matched male rats compared to their younger female and male counterparts. This effect was accompanied by reduced hippocampal expression of claudin-5 only in the senescent females (expression in the thalamus was not reported). These results indicate that, while ageing impairs BBB functioning in both sexes, this effect may be linked more strongly to changes in the expression of tight-junction proteins in the hippocampal BBB of female rats. However, Frías-Anaya [204] found that age-related capillary changes, that could degrade BBB functionality, were larger in the cortex than in the hippocampi of female mice. 

These findings indicate that the breakdown of the BBB is a prominent aspect of aging, and reports from diverse labs suggest this is observed more prominently in the hippocampus compared to other areas of the brain. However, it can be difficult to separate the harmful effects of aging on the hippocampal BBB from the impacts of other age-related factors, which are often unaccounted for and could interact with the effects of aging. For example, Tucsek and her collaborators [205] compared 7- and 24-month-old male mice that had been maintained on an obesity-inducing, high-fat diet or standard, low-fat laboratory chow. BBB permeability in the hippocampus was increased, and the expression occludin and claudin-5 reduced in the older mice. These effects were exacerbated in mice that became obese on the high-fat diet. Moreover, in humans, there may be many variables that are experienced early in life that could accelerate or intensify the detrimental effects of aging on hippocampal-dependent cognitive function and the hippocampal BBB [206,207].

Aging—Conclusions: In summary, there is clear evidence that aging adversely impacts the hippocampus and the hippocampal BBB. Some data also suggest that the effects of aging may appear first and more prominently in the hippocampus. There are also data that are consistent with the possibility that BBB disruption in aging also occurs first and foremost in the hippocampus, but this question is not yet settled. 

### 2.6. Neurodegenerative Disease

Two diseases, Alzheimer’s (AD) and Parkinson’s (PD), were selected based upon them being, respectively, the first and second most prevalent neurodegenerative conditions [208]. 

#### 2.6.1. Alzheimer’s Disease (AD)

AD is characterized by progressive impairment of episodic memory, and more broadly with cognitive decline, physical disability, and ultimately death [209,210]. The early involvement of episodic memory is important, as this links it to hippocampal integrity [211,212,213]. Damage to the hippocampal formation seems to be a central and fundamental aspect of this disease, with AD even labelled as a hippocampal dementia [214]. The entorhinal cortex [215] and the adjacent hippocampal structures [211] are clearly implicated in AD. The changes observed to the hippocampus are distinct from those of healthy aging [216]; indeed, hippocampal damage—as evidenced via volume loss—is so typical of AD that the European Medicines Agency selected this structure as the first MRI biomarker for use in AD-related clinical trials [217].

While there is little argument about the involvement of the hippocampus in AD, an important remaining question is whether it is the first site of damage. One approach is to look at the predictors of conversion from mild cognitive impairment (MCI; a prodromal phase) to AD. While brain MRI markers, including hippocampal volume, are predictive, so are many other variables (e.g., dementia rating scales, serum biomarkers, etc. [218]). This suggests that MCI may be too late in disease progression to isolate the initial change. Another approach is to study asymptomatic people who have a genetic predisposition (typically APOE4) for sporadic AD. Carriers of APOE4 have greater rates of hippocampal atrophy across time than non-carriers [219]. While cardiovascular risk factors probably account for some of these brain changes, evidence for hippocampal abnormality in asymptomatic carriers has still been observed [220]. A recent analysis of MRI findings appears to be more definitive. Planche et al. [221] combined multiple large-scale databases comprising 3512 quality-controlled whole-brain MRI scans from nine cohorts of subjects at different ages covering the entire lifespan—including 415 scans of AD patients—to create lifetime volumetric models of brain structures which are with and without Alzheimer’s disease. This analysis revealed a chronological progression of brain atrophy that began with the hippocampus and amygdala, moving next to the middle temporal gyrus, then to the entorhinal and other temporal cortex areas, followed by the striatum and thalamus, and finally pallidum and the middle frontal, cingular, parietal, and insular cortices. This pattern of atrophy was like that described by Braak and Braak [222] based on post-mortem samples, except the hippocampus replaced to entorhinal cortex as the initial site of disease pathology. In summary, there is evidence that favors the hippocampus both as an early and possibly first structure impacted by AD, and as a structure that is possibly most affected over the course of the disease. 

AD and the BBB: A number of findings also show that the permeability of the hippocampal BBB is significantly increased in humans diagnosed with AD [223] and in rodent models of this disorder [224]. For example—in addition to observing the previously mentioned selective increases in hippocampal BBB permeability as a direct function of age—Montagne et al. [15] reported that, compared to age-matched adults with normal cognitive functioning, breakdown of the hippocampal BBB was greater for people diagnosed with mild cognitive impairment and greater still for people diagnosed with AD. This pattern of results was also obtained by Zhang et al. [223] who used diffusion MRI to assess the BBB permeability to water molecules without using a contrast agent. Other evidence indicates that both increased BBB permeability and decreased cognitive function are associated with damage to capillary pericytes in the human hippocampus (see [205] for a review). Using a novel biomarker of BBB-associated pericytes in a large cohort (N = 161) of adult participants, Nation et al. [225] showed that the progressive damage of pericytes was significantly associated with impaired cognitive functioning. Moreover, individuals with early cognitive dysfunction exhibit BBB breakdown in the hippocampus irrespective of levels of Aβ1-42 or tau protein (long considered biomarkers for Alzheimer’s disease). Furthermore, in a subset of 73 participants, DCE-MRI analysis revealed increased BBB permeability in the total hippocampus, and the CA1, CA3, and dentate gyrus subfields and in the parahippocampal gyrus in cognitively impaired relative to unimpaired participants. Consistent with the study by Montagne et al. [15], cited above, BBB permeability differences between the cognitively impaired and unimpaired groups were not found in frontal and temporal cortex, subcortical white matter, corpus callosum, or internal capsule, nor in deep gray matter regions, including the thalamus and the striatum. As was the case with the biomarkers of pericyte damage, BBB breakdown and cognitive impairment were not associated with elevated CSF levels of Aβ1-42 or tau in this cohort. Considered together, these findings support the view that BBB breakdown and/or dysfunction is a precursor to the dementia and neurodegeneration that is symptomatic of Alzheimer’s disease [16]. 

However, not all findings have been consistent with the view that BBB breakdown is an early and prominent feature of AD. For example, Chiquita and colleagues [226] reported that BBB leakage was only a late consequence in an AD mouse model, emerging well after other disease symptoms. Also, Bien-ly and collaborators [227] tested several different mouse AD models and found no evidence of increased BBB permeability. However, the extent to which mouse models of AD recapitulate all features of the disease, including BBB breakdown, can be questioned [228,229]. Whether these results reflect a limitation of these models or are a cautionary note about the involvement of the hippocampal BBB in AD remains to be decided.

There is also a genetic link between AD and BBB disruption. Carriers of the APOE4 gene, which is the strongest genetic risk factor for Alzheimer’s disease [230,231], are more susceptible to the accelerated degeneration of pericytes and BBB breakdown compared to people without this gene variant [232,233]. Moreover, Montagne and his group [234] reported that BBB breakdown in APOE4 carriers is most pronounced in the hippocampus and medial temporal lobe; while this breakdown was more severe in patients with cognitive impairment, it was also observed in cognitively unimpaired APOE4 carriers. Further, consistent with earlier findings [15], this BBB disruption associated with the APOE4 gene was not found to be related to amyloid-β or tau pathology. In addition, Montagne et al. [234] reported that higher levels of a biomarker for BBB pericyte damage predicted future cognitive impairments for APOE4 carriers, but not in non-carriers. 

The association between BBB disruption and impaired hippocampal function may be based, in part, on disturbances in glucose transport (see [235] for a review). Glucose is the main fuel source for mammalian brains [236], and it depends on the glucose transporter GLUT1 to cross the BBB from the bloodstream into the extracellular space [237,238], whereas another transporter, GLUT3, facilitates glucose uptake by neurons [239]. In addition to its role in transporting glucose into the brain, brain capillary networks and BBB integrity also depend on GLUT1 [240]. Because neurons are typically intolerant of energy deficits, a number of brain pathologies (e.g., oxidative stress) may be the consequence of reductions in glucose transport across the BBB [236]. The initial stages of AD are accompanied by both diminished GLUT1 expression at the BBB and reduced glucose uptake; these conditions become worse with disease progression [240]. Reduced glucose uptake in the hippocampus and some cortical areas have been revealed by fluorodeoxyglucose-positron emission tomography (FDG-PET) scans in humans with early-stage AD, mild cognitive impairment, and with genetic or other risk factors for AD, even in the absence of cognitive sequelae [225,241,242]. Changes in FDG-PET that precede evidence of neurodegeneration have also been found in transgenic mouse models of AD [243]. In addition, mouse models also indicate that reduced GLUT1 precedes increased BBB permeability with both events increasing AD pathophysiology [244]. Other findings suggest that NVU dysregulation, which includes impaired endothelial transport, pericyte degeneration, and a leaky BBB, also exacerbates AD pathogenesis. This occurs by reducing Aβ clearance across the BBB, thereby resulting in greater Aβ accumulation in the brain [245]. The preceding analysis indicates that the hippocampal BBB may be the first and is possibly the most prominent site of damage in AD.

#### 2.6.2. Parkinson’s Disease

PD is characterized by its clinical motor symptoms resulting from damage to the nigrostriatal pathway [246]. There are also cognitive impairments arising from damage to striatal–frontal lobe projections [247,248]. As with other neurodegenerative disorders, adverse changes to the brain are likely to predate the first clinical signs [249]. Post-mortem staging reveals that the earliest signs of the disease occur in the olfactory bulb and in the autonomic and enteric nervous systems [250]. Progression occurs from the brain stem to the mid-brain and then to the neocortex. PD does not involve a core role for the hippocampus, although this structure is affected later in the disease [251]. Indeed, hippocampal damage may contribute to cognitive impairment [252] and PD dementia, the latter characterized by Lewy pathology in this structure [252,253]. To summarize, PD does not involve initial or primary hippocampal pathology. 

PD and the BBB: There appear to be no reports linking Parkinson’s disease with disruption in the functioning of the hippocampal BBB.

AD and PD—Conclusions: In AD, the hippocampus plays a primary and prominent role in the disease and is likely to be the initial site of damage. Similarly, there is evidence that BBB disruption occurs first and is most pronounced in the hippocampus. The hippocampus does not play a significant role in PD and whether or not PD disrupts the hippocampal BBB has not been investigated. 

### 2.7. Acquired Brain Injury

This section examines two forms of acquired brain injury. The most common is stroke [254], but its heterogenous nature (location, type, and severity) make it difficult to evaluate. For this reason, we focus instead on cerebral ischemia and hypoxia, which is common and can result from cardiac arrest, shock, drug overdose, and certain types of brain injury [255,256]. The second focus is on mild traumatic brain injury (TBI) and its sequelae [257]. Mild TBI is also common (150 million cases/year).

#### 2.7.1. Cerebral Ischemia and Hypoxia

Human case studies of ischemia/hypoxia have been theoretically important because of their hippocampal selectivity, playing a key role in demonstrating the importance of the hippocampus to declarative memory [258,259]. Post-mortem data suggest that the CA1 layer of the hippocampus is highly sensitive to the effects of ischemia/hypoxia, probably more so than any other brain area [255,260,261]. Ischemia/hypoxia causes significant decrements in performance on tests of HDLM, more so than other cognitive domains [262]. The effect of ischemia/hypoxia in humans seems to parallel the effect in animals [263]. This is important because of the greater control that can be exerted over the ischemic/anoxic episode [264,265]. Even brief episodes of ischemia/hypoxia can produce both immediate damage to the CA1 layer of the hippocampus and delayed cell death [266]. Extending the period of ischemia/hypoxia produces progressively greater damage to the brain, in a fairly ordered way, with the neocortex and then the striatum being affected [264,266]. Experience of hypoxia/ischemia is also a known risk factor for AD [267]. In sum, hippocampal damage occurs first and foremost in ischemia/hypoxia.

Ischemia/hypoxia and the BBB: Ischemia/hypoxia is associated with BBB disruption in both experimental animal models and in human stroke patients [268,269], with sustained ischemia producing increased BBB permeability [270,271] and reperfusion following ischemia causing further barrier damage [272,273,274]. In a study designed to investigate the effects of hypoxia that occurs as a consequence of cardiopulmonary bypass (CPB) surgery [275], Lui et al. reported that, following recovery from a 2 h CPB operation, rats exhibited markedly impaired hippocampal-dependent performance in the Morris water maze, increased permeability of the hippocampal BBB, along with elevated levels of proinflammatory cytokines in hippocampal tissue. There is also evidence that an inflammatory response, marked by elevated levels of TNF-α and IL-1β, is activated within hours after reperfusion [276]. This contributes to weakening of the BBB [272,277]. In addition, as described for epilepsy, BBB damage in ischemia involves the upregulation of MMPs [278], resulting in the degradation of the tight junctions between endothelial cells and the disturbed binding of astrocyte end feet to the vascular wall [279]. 

Several studies have reported that ischemia is associated with selective disruptions of the hippocampal BBB in the CA1 region [280,281], whereas the CA3 region is largely spared [282], a pattern that coincides with the neuronal degeneration that is seen in the same areas [283,284]. In another study, Lee et al. [285] assessed BBB permeability differences in the CA1 and CA3 regions of the gerbil hippocampus following mild and severe ischemia produced by carotid artery occlusion for 5 min and 15 min, respectively. BBB leakage was of greater magnitude and occurred more quickly in the CA1 region following 15 min, compared to 5 min ischemia. Ischemia/hypoxia can also have a long-lasting impact on BBB functioning. One year after ischemia/hypoxia produced by transient middle cerebral artery occlusion, BBB permeability in rhesus monkeys was significantly higher, compared to age-matched controls, in the caudate nucleus, white matter, and thalamus. However, the biggest increase was observed in the hippocampus [286]. Relative to other brain areas, it appears that disruptions in the protection provided by the hippocampal BBB occur earlier and may be a more prominent contributor to the adverse effects of ischemia/hypoxia on this structure.

#### 2.7.2. Traumatic Brain Injury (TBI)

In humans, mild TBI can result in a range of cognitive impairments to memory, attention, and processing speed, which usually resolve quickly [287,288,289]. Neuroimaging of people following a single mild TBI can often reveal no abnormalities, although there are reports of reduced activity in the default mode network and increased hippocampal activity on functional imaging [290]. With structural imaging, reductions in hippocampal volume are far more evident if the person has experienced repeated mild TBIs [291], and possibly to a greater extent than for other brain areas [292]. There is also an emerging association between mild TBI and increased risk for AD [293]. The strongest evidence for hippocampal vulnerability following mild TBI comes from animal data using the lateral fluid percussion (FP) method [294]. Using the mildest force (noting that the effects are cumulative if the injury is repeated [294,295]) produces little cell death in the cortical tissue directly receiving the blow. However, it results in multiple hippocampal-related abnormalities in the days and weeks following this injury [294,296,297]. While other brain areas are also sensitive to this procedure (cortex, amygdala, striatum, and thalamus), it is the consistency of hippocampal deficits and the low level of injury needed to provoke them which mark out this structure as especially vulnerable in animal models. In summary, human and nonhuman animal data are divergent. While the nonhuman animal data point strongly to the hippocampus being the most affected site (but not the primary site), the human data are more equivocal. However, if there is repetitive mild TBI, then differential damage to the hippocampus becomes more evident.

TBI and the BBB: It is well established that the hippocampal BBB of rodents is disrupted following mild TBI, but it is not the only site of BBB damage [298,299]. For example, rats exposed to repetitive mild TBI over a 2-week period exhibited significantly increased BBB permeability in both the cortex and hippocampus relative to controls that had not received repeated sub-concussive impacts [300]. Another study [301] observed increased BBB leakage that peaked 4–6 h after controlled impact injury in rats, before a second opening occurred after 3 days post-injury, both at the cortical contusion site and in the ipsilateral hippocampus. A biphasic response was also reported by Logsdon et al. [302] who examined the effects of repetitive (2x) mild blast exposure on the BBB permeability of mice in the frontal cortex, occipital cortex, parietal cortex, striatum, midbrain, thalamus, cerebellum, pons/medulla (brainstem), and hippocampus. Based on extravasation of a relatively small radiotracer (C-14 sucrose) or a larger molecular weight radio tracer (99mTc-albumin), repetitive blast exposure elicited BBB permeability increases at 15 min and at 72 h, but not at 24 h post-injury, which were most pronounced in the frontal cortex and hippocampus for both tracers. In summary, each of the above studies reported that, relative to other brain areas, increased BBB permeability in both the hippocampus and the cortex are earlier and more prolonged consequences of mild TBI. 

Ischemia/Hypoxia and TBI—Conclusions: In ischemia/hypoxia, hippocampal damage occurs first and is more pervasive compared to other structures and the timing and the extent of this damage corresponds to the disruption that takes place in the hippocampal BBB. For mild TBI, while animal data indicate differential hippocampal pathology following a single insult, the human data suggest that multiple mild TBIs may be necessary for this to become evident. TBI is also associated with rapid and sometimes biphasic increases in BBB permeability that affect the hippocampus and other brain areas.

### 2.8. Mental Health Conditions

Depression and anxiety are the first and second most prevalent mental health disorders [303]. For depression, we examine the commonest form, namely major depressive disorder (MDD). For anxiety, which is composed of several disorders, we focus on the most generic, namely generalized anxiety disorder (GAD) [304].

#### 2.8.1. Major Depressive Disorder (MDD)

There are several major theories of MDD. These have been organized based on their degree of involvement of the hippocampus, from most to least relevant. The most relevant theory is the neurogenesis account [305]. This has two central ideas. First, reduced neurogenesis in the dentate gyrus causes MDD. Second, medications and behavioral interventions that remedy MDD exert their therapeutic effect by reinstating neurogenesis. The first contention has not withstood study in animals, as various manipulations designed to reduce neurogenesis do not cause depression-like symptoms [306]. The second contention is better supported and fits especially neatly with the therapeutic delay observed for anti-depressant medications [307,308], for behavioral interventions [306], and for the beneficial effects of electroconvulsive therapy [309].

A second theory, which overlaps with the first, concerns stress and inflammation. Chronic stress adversely affects the hippocampus, reducing its volume—as observed in people with MDD [310,311,312,313]. As the hippocampus is a major part of the feedback loop that reduces a stress response, chronic stress may impair this, leading to consistently elevated cortisol levels—again, as observed in MDD [314]. This in turn may lead to chronic inflammation, which can also generate many MDD-like symptoms. Neurogenesis is impaired in this model as part of the broader impacts of stress on the hippocampus, with anti-depressant medications exerting their effects on the inflammatory response [315].

Other theories have developed around the idea of interactive control of limbic emotion circuits by frontal structures [316]. These theories are multi-centered/-network models; hence, the role of particular brain structures is deemphasized. The hippocampus has a less defined role here, with R. Davidson [317] suggesting it exerts contextual control when depressive behaviors are expressed, and its dysfunction leads to a failure of contextual control and the demonstration of such behaviors (sadness, fatigue, etc.) in all contexts. 

Finally, there are theories that have only a very limited role for the hippocampus. The monoamine hypothesis is one [318], although more recent models do draw in subcortical structures [319]. Similarly, there are both circadian [320] and interoceptive theories of MDD [321]; while the hippocampus arguably plays a role in interoceptive processing, neither suggests a dominant hippocampal role.

In summary, MDD is associated with hippocampal impairment, and MDD is also a risk factor for AD [322]. Some theories suggest hippocampal dysfunction is the cause of depression, others relegate it to an effect—major or minor. It is unlikely that any one brain area or theory can explain all cases of MDD. However, it seems that, for some cases of MDD, especially those involving a major stress response, the hippocampus will have a causal role and be the most adversely affected structure. 

MDD and the BBB: The results of studies using rodent models provide evidence that increases in several behavioral indices of depression (e.g., learned helplessness, anhedonia, decreased mobility, reductions in escape behavior) induced by stress are linked to the breakdown of the hippocampal BBB. Following exposure to chronic mild stress, Taler et al. [323] used DCE-MRI to assess BBB permeability in five brain regions (hippocampus, nucleus accumbens, prelimbic and infralimbic cortices, and frontal associative cortex) related to depression. In this study, increased BBB permeability was confined to the hippocampus; however, other studies employing other stressors and measuring extravasation of substances across the BBB have reported that permeability was also increased in the prefrontal cortex [324,325,326]. BBB disruption has also been indicated by findings of reduced levels of tight-junction proteins, including claudin-5, zona occuldins-1 and occludin in the hippocampal BBB [324,325,327]. 

In addition to the results from rodents, Greene et al. [328] made post-mortem comparisons of seven brain areas (parietal cortex, occipital cortex, cerebellum, premotor frontal cortex, and caudal cingulate cortex, orbitofrontal cortex, and hippocampus) from patients who had been diagnosed with schizophrenia, bipolar disorder, or MDD or were controls with no known brain disorder. Claudin-5 levels in the hippocampus were significantly reduced in the patients with depression and schizophrenia, with no differences found in the other brain regions or patient groups. 

Considered together, the above findings suggest that disruption of the hippocampal BBB may have a key role in the manifestation of MDD in humans. However, one can question the generalizability of findings from rodent models of chronic-stress-induced depression-linked behaviors to the more complex etiology and behavioral expressions of human MDD. This, along with the lack of studies investigating links between MDD and the functioning of the human hippocampal BBB, make stronger conclusions difficult.

#### 2.8.2. Generalized Anxiety Disorder (GAD)

While hippocampal pathophysiology plays a role in GAD [329,330], the main drivers are the amygdala and frontal circuits [331,332,333]. This pathophysiology mirrors contemporary theorizing about the origins of GAD in abnormal fear responses (amygdala) and abnormal modulation of this response (frontal circuits). Interestingly, anxiety has been found to be an independent risk factor for AD [334]. In sum, the hippocampus is abnormal in GAD, but it is neither the first nor most affected structure.

There appear to be no studies that have explicitly investigated potential links between BBB disruption and GAD in humans. Moreover, it is difficult to disentangle anxiety from depression, experimentally, in nonhuman animal models. Those models typically rely on exposing rodents chronically to uncontrollable or unpredictable aversive stimuli or conditions, with the suppression of often the same behavioral responses serving as the index of anxiety, depression, or both [335,336]. Accordingly, our preceding discussion of MDD in relation to the functioning of the hippocampal BBB is presumably also relevant to GAD, with the same limitations applying to both disorders.

MDD and GAD—Conclusions: Some cases of MDD are probably caused by hippocampal pathophysiology, with this structure being damaged most. In other cases, the hippocampus may play a subsidiary role, as MDD is a heterogenous disease. The available findings also suggest that disruption of the hippocampal BBB may contribute to the manifestation of MDD in humans, relative to the BBB in other brains areas. While nonhuman animal models of MDD are also consistent with this possibility, the translation of results from rodents exposed to chronic stress to the more complex etiology and behavioral expressions of human MDD can be questioned [337]. For GAD, while there is hippocampal pathology, it is not first or foremost and there are insufficient data on which to base conclusions about the relationship of GAD to BBB functioning.

### 2.9. Endocrine Disorders

The two commonest endocrine disorders are diabetes and thyroid dysfunction [338,339]. For diabetes, we focus on the most common: Type 2 adult-onset form [339,340]. For thyroid dysfunction, we focus on the most common manifestation—hyperthyroidism [338]. 

#### 2.9.1. Diabetes

Two lines of evidence suggest that Type 2 diabetes (T2D) significantly impacts the hippocampus. First, neuropsychological deficits are evident in all domains [341,342], but especially in tests of HDLM [343]. Second, meta-analysis of structural MRI studies contrasting T2D patients and controls, reveals multiple abnormalities, with an overall reduction in brain volume, and regional atrophy in the hippocampus and other areas [344]. However, hippocampal atrophy is the largest regional abnormality [345]. One complication of comparing T2D patients with controls, is the presence of comorbid conditions (hypertension, obesity, depression, dyslipidemia) that could account for brain differences. However, even when these conditions are controlled, hippocampal atrophy is still related to T2D [346,347]. It is noteworthy that T2D elevates risk for AD and MDD [348,349], also potentially implying hippocampal involvement.

Four sets of findings suggest that the hippocampus may be the first structure damaged in T2D. First, early in the disease HDLM impairments, accompanied by hippocampal atrophy, are evident before those of other domains [347]. Second, lower glucose tolerance is associated with poorer HDLM performance and smaller hippocampal volume in healthy, nondiabetic controls. These relationships were observed even before the formal onset of T2D and hippocampal impairment was evident [350]. Third, in a meta-analysis of structural imaging studies [344], one key effect was noted. For humans with T2D, only the hippocampal region was sensitive to sample age, with the largest difference relative to controls evident in the youngest samples. This also suggests that hippocampal deficits appear early in T2D. Fourth is the nonhuman animal literature, with its excellent control of comorbidities. This finds rapid reductions in hippocampal neurogenesis [351], abnormal hippocampal morphology, and impaired function [351], suggesting that the hippocampus is an early target of this disease. In sum, it seems likely that the hippocampus is the first brain region affected by T2D and may be the most affected.

Diabetes and the BBB: Several reports indicate that BBB permeability is increased in nonhuman animal models of T2D [352,353] and imaging studies have shown that human Type 2 diabetics exhibit increased permeability of the BBB compared to nondiabetic controls [354]. The effects of diabetes on the integrity of the hippocampal BBB have also been investigated. For example, in a study by Yoo et al. [355], diabetes-prone Zucker rats showed significantly greater hippocampal BBB permeability and significantly reduced expression of occludin and claudin-5 compared with nondiabetic control rats. In addition, using the mutant db/db mouse model of T2D, Nuthikattu et al. [356] performed transcriptomic analyses in diabetic hippocampal endothelial cells and gadolinium contrast enhanced MRI to assess BBB permeability in the whole brain and the hippocampus. Compared to wild-type controls, the transcriptome of the diabetic mice revealed a pattern of gene expression changes in hippocampal endothelial cells that were indicative of dysfunction (e.g., disruption of cell–cell adhesion; disordered formation of tight junctions). These observations were accompanied by greater gadolinium signaling in the hippocampus of the diabetic rat, confirming greater hippocampal BBB permeability, relative to controls.

Other data indicate that the deleterious effects of T2D on the BBB extend beyond the hippocampus. For example, using DCE-MRI, it was found that BBB permeability was dramatically increased in the hippocampus of diabetic rhesus monkeys, but to no greater extent than for the BBBs of the frontal cortex, temporal cortex, basal ganglia, and thalamus [357]. Another report showed that permeability was significantly higher in the thalamus and white matter of humans diagnosed with T2D compared to nondiabetic participants, whereas this difference was not significant for the hippocampal BBB [358]. However, as the authors noted, the interpretation of the findings is complicated by several issues: (a) of the 114 participants in the study, only 7 people were in the diabetic group; (b) the mean age of the participants was 67.3 +/− 0.5 years, raising the possibility that the effect of aging may have been greater on hippocampal BBB permeability compared to the other brain areas. Overall, the data indicate that the hippocampal BBB is damaged by T2D but there is little agreement among about whether it is damaged first, or more extensively, compared to the BBBs of other parts of the brain.

#### 2.9.2. Hypothyroidism

People with clinical hypothyroidism often report cognitive impairments, especially for learning and memory [359,360]. When tested before treatment, they reveal relatively selective impairments in HDLM [361], and after treatment, thyroxine performance improves [361,362]. It was originally suspected that clinical hypothyroidism was a risk factor for AD, but two meta-analytic reviews do not support this [363,364]. A report by van Vliet et al. [364] also found no evidence of memory impairment. This finding is problematic as it is based on combining tests of short- and long-term memory, which may mask selective deficits to HDLM. HDLM deficits should also be expected, as there is strong evidence that thyroid hormones play multiple key roles in hippocampal function [365,366]. Indeed, neuroimaging finds reduced hippocampal volume in patients with hypothyroidism [367,368,369,370], although not all studies observe this [371] and atrophy in other regions is also seen (e.g., amygdala, frontal cortex, cerebellum [367,369,370]). Metabolic abnormalities have also been identified in several regions, including in the hippocampus, in frontal and cingulate cortices, and in the cerebellum [368]. A further reason to suspect hippocampal involvement comes from rodent studies. Experimental induction of hypothyroidism produces a rapid reduction in hippocampal neurogenesis [372,373]. This is accompanied by selective impairments on tests of HDLM—effects that are well established and replicable [374]. In sum, for humans, there is good evidence for hippocampal impairment in clinical hypothyroidism, but it is unclear if this is first or foremost. In contrast, nonhuman animal data suggest that the hippocampus is affected first and foremost.

Hypothyroidism and the BBB: There is relatively little information concerning the relationship between hypothyroidism and the integrity of the BBB in humans or nonhuman animals. Furthermore, there appears to be no studies that have examined this relationship as it pertains to the hippocampus. Two studies have investigated the effects of chronic canine hypothyroidism on the integrity of the BBB. In one study, iodine-induced hypothyroidism in dogs was accompanied 12 and 18 months later by elevated levels of VEGF and albumin in cerebrospinal fluid (CSF), two biomarkers linked to BBB dysfunction [375]. The second study [376] assessed whether chronic canine hypothyroidism was also associated with increased CSF levels of the MMPs and endothelin-1 (a marker of ischemic brain damage). No significant differences were found relative to control animals. 

Diabetes and Hypothyroidism—Conclusions: Data from both human and nonhuman animals suggest that the hippocampus is the first brain region to be affected by T2D, as well as possibly the most affected. In addition, much, but not all, of the data indicate that the hippocampal BBB is highly sensitive to disruption by T2D, and it may be disrupted earlier and more prominently compared to other brain areas. For human clinical hypothyroidism, there is good evidence for hippocampal impairment. However, while nonhuman animal data strongly indicate that the hippocampus is affected first and foremost, it is currently unclear if this is the case for people. There are little data available on which to assess whether deterioration of hippocampal functions in hypothyroidism involved changes in the functions to the hippocampal BBB.

### 2.10. Developmental Disabilities

Two types of developmental disability are examined. The first is Down syndrome (DS), which results from abnormal duplication of chromosome 21 and is the commonest form of cognitive disability [377]. The second is autism spectrum disorder (ASD), which is included as it affects around 52 million people worldwide [378]. 

#### 2.10.1. Down Syndrome (DS)

The DS brain demonstrates multiple abnormalities, many of which may link to impaired GABAergic signaling [379]. As GABAergic neurons are widespread [380], this type of theory does not lend itself to identifying structure-specific abnormalities. Nonetheless, structural differences are evident, with a smaller brain, and an abnormal cerebellum, frontal cortex [381], and hippocampus [382]. Hippocampal abnormalities are especially marked in the well-studied Ts65Dn mouse model of DS [383], as are hippocampal connections to other brain regions [384]. These deficits closely parallel behavioral deficits in people with DS, who have particularly poor HDLM, but are intact on several other forms of memory that are not hippocampal-dependent [385]. Indeed, this distinctive pattern of memory impairment is so characteristic of DS that it is included in the standard neuropsychological battery for developmental disabilities (Arizona Cognitive Test Battery; ACTB). Lest this give undue emphasis to the hippocampus, it is important to note that tests for frontal and cerebellar function are also included in the ACTB, because of their known sensitivity for DS-related cognitive deficits. In sum, while hippocampal abnormalities are a prominent in DS—including the very high risk for Alzheimer’s-like dementia [386]—they are not first and probably not foremost.

DS and the BBB: There appear to be no studies that provide evidence that DS affects the integrity of the hippocampal BBB directly. There is evidence that genes on chromosome 21 are associated with disorders that can adversely affect BBB integrity and which associate Down syndrome with increased incidence of AD [387]. Although AD and DS have similarities in their genetics and symptom onsets, the role of BBB disruption and other neurovascular impairments in DS has received little attention [388].

#### 2.10.2. Autism Spectrum Disorder (ASD)

Although ASD is a heterogenous condition, there is some agreement over its neuropathology. ASD has a significant genetic component, which leads to multiple abnormalities in the developing brain [389], notably to the neocortex [390], and in the function of the GABAergic system [391]. Structurally, toddlers with ASD often have larger brains, greater cortical area, and abnormal gyrification [392]. Several brain areas/features have been identified as atypical in ASD [390]. These include the amygdala, the lack of cortical asymmetry, the cerebellum, corpus callosum, striatum [393], and hippocampus [394]. While the hippocampus is often enlarged in ASD, it does not appear to be either the first or most affected structure, and there is no evidence linking ASD to risk for sporadic AD. 

ASD and BBB: Prenatal maternal infection has been identified as a risk factor for childhood ASD. Garay et al. [395] modeled this risk factor by inducing immune activation in pregnant mice and then measuring cytokine levels and BBB permeability in the progeny at several time points from birth to 60 days of age. While altered (typically elevated) levels of proinflammatory cytokines were observed in the postnatal hippocampus, frontal cortex, and cingulate cortex, no breaches of the BBB in any of these brain areas were observed. Exposure during pregnancy to valproic acid (also known as, valproate), a treatment for epilepsy and other neurological disorders, increases the risk of postnatal ASD in human children [396]. Deckmann et al. [397] measured BBB function following prenatal exposure to valproic acid (VPA) to model ASD in mice. Compared to controls, VPA-treated mice exhibited BBB permeability to Evans blue dye in primary somatosensory area, medial prefrontal cortex, and other neocortical areas, but not in the hippocampus. Thus, at present, there is no evidence that links ASD to alterations in the hippocampal BBB.

DS and ASD—Conclusions: Hippocampal abnormalities occur in both DS and ASD, but they are not first or foremost in either disorder. The role of the BBB in DS has received little research attention. Findings from studies of rodent models of ASD have obtained no evidence of an effect on the BBB. 

### 2.11. Nutrition

Obesity and obesogenic diets that contain elevated levels of added sugar, salt, and saturated fat (e.g., Western diets (WD)) are both widely consumed in Western and Westernized societies and are associated with increased prevalence of many undesirable health consequences [398,399]. In contrast, globally in 2022, 149 million children <5 y of age were estimated to be stunted (too short for their age) and 45 million were estimated to be wasted (too thin for their height) as a consequence of undernutrition defined by deficiencies in nutrient intake, imbalances in the intake of essential nutrients, or impaired nutrient utilization [400]. The effects of obesity and obesogenic diets and undernutrition on the hippocampus and the BBB will be considered here.

#### 2.11.1. Obesity and Obesogenic Diets

Several studies have examined the links between brain function/structure and adiposity. Establishing this relationship requires adequate control for confounding variables (e.g., T2D, socioeconomic status [401,402]). Neuropsychological testing, with appropriate controls, suggests that deficits in speed of processing, executive function, inhibitory and reward processes, and HDLM are all linked to adiposity [403]. Neuroimaging indicates that reward-related (e.g., striatum) and executive/inhibitory control structures are correlates of adiposity [404]—as well as the hippocampus—when controlling for confounders [405,406]. A significant issue here is discriminating between preexisting neural risk factors for adiposity and its consequences [407]. A key approach is to see whether genetic risk factors for obesity moderate the relationship between brain structure and adiposity. Weise et al. [408] found that the strongest moderation of structure–adiposity relationships was for the cerebellum, amygdala, putamen, and nucleus accumbens. Weaker moderation was observed for the hippocampus and insula. Weise et al. suggested that this pattern arises because changes in these latter structures are a consequence of adiposity. This is consistent with nonhuman animal work, which finds multiple mechanisms capable of instantiating the effect of excess adipose tissue specifically on the hippocampus [409,410]. Thus, while there is some evidence that the hippocampus is adversely affected by adiposity, it cannot be concluded that it affected first or more prominently compared to other brain structures. 

There is, however, an important additional consideration. In many studies, it is difficult to disentangle the effects of obesity on the brain from the intake of diets that produce obesity. For example, diets that are high in fat or high in fat and sugar (also known as a Western-style diet (WD)), are considered to be major contributors to weight gain, obesity, and their assorted co-morbidities in humans [399,411,412]. In addition, these types of diets are commonly used to produce excess body weight and adiposity in rodents [413,414]. In these studies, the association between obesogenic diets and selective hippocampal dysfunction appears to be strong. For example, a review and meta-analysis of rodent data indicated that hippocampal dependent spatial learning and memory is impaired—sometimes in a matter of days—by consumption of a WD [415]. In addition, several reports showed that the performance of rats maintained on ad libitum WD were impaired, relative to controls fed standard chow, on nonspatial discrimination problems that depended on the integrity of the hippocampus, but not on discriminations that are hippocampal-independent [416,417,418]. While other brain structures can also be affected, notably the prefrontal cortex [419], this does not occur with the same rapidity or consistency. Human studies find a correlation between eating a WD and poorer hippocampal function and show that exposure to a WD can cause hippocampal dysfunction within a few days for lean people who normally eat a healthy diet [420,421]. WD also affects other brain regions, but hippocampal effects are early and robust, as identified in a recent systematic review and meta-analysis [422]. In summary, the hippocampus appears to be the first and major target of disruption produced by consuming a WD. 

Obesity/obesogenic diets and the BBB: There are many findings linking obesogenic diets to disruption of the BBB in rodents. Factors such as diet composition (e.g., 40–60% saturated fat), duration of diet exposure (days–months), species (e.g., mouse, rat, primate), age (juvenile–adult), and measures of BBB function (e.g., extravasation of different substances, MRI, expression of transporters, and tight-junction proteins) have differed across studies. Nonetheless, BBB disruption has been a common outcome for animals fed a WD or a similar energy-rich diet. For example, increased hippocampal BBB permeability has been reported in many studies [205,423,424,425,426,427,428,429]. WD consumption has also been associated with reductions in tight-junction proteins in the hippocampal BBB [417,423,427,429,430,431,432]. Furthermore, reduced expression of transporters for energy metabolites (e.g., GLUT1, MCT1) and hormones known to promote memory function (e.g., leptin) at the level of the hippocampal BBB has also been reported [409,417,423,427,431,433,434,435,436]. 

There is also evidence that obesogenic diets increase BBB permeability in the hippocampus, while leaving the BBB in other brain areas largely unaffected. For example, compared to chow-fed controls, rats maintained on WD for 90 days exhibited increased extravasation of NaFl in the hippocampus but not in the striatum or prefrontal cortex [417]. Similarly, mice fed an obesogenic diet for 36 weeks showed significant BBB permeability in the hippocampus, as indexed by the level of infused radioactive albumin (a blood-borne protein that does not cross the BBB under normal conditions), but not in the frontal, parietal, or occipital cortices or the thalamus or midbrain [424]. Furthermore, increased BBB leakage in the hippocampus, but not in the prefrontal cortex, striatum, or cerebellum, has been reported for rats that became obese on a WD compared to chow-fed controls [418]. Notably, significantly increased BBB permeability was not observed for “diet-resistant” (DR) rats that were fed with a WD but did not gain significantly more weight than controls. It is not clear whether the differences in body weight or in amount of WD consumed by obese and DR rats accounts for this result. In addition, Hargrave et al. [426] found that, compared to either DR rats fed WD or chow-fed controls, rats that became obese on a WD exhibited greater BBB permeability in the total hippocampus and in many of its subregions (CA1-3, dentate gyrus, subiculum), with a smaller increase in the dorsal striatum, but with no differences in the total or ventral striatum or in the cerebellum. The findings of increased BBB permeability differences were obtained following 90 days on a WD, whereas no significant differences were observed after either 10 or 40 days. 

BBB leakage has also been reported following short durations of exposure to an obesogenic diet. In one experiment [428], mice maintained on a diet with a higher fat content (60%), compared to the diet given to rats in the Hargrave et al. study (~40%), exhibited increased hippocampal BBB permeability following one day of diet access, before returning to baseline levels after 4 days. A second experiment in the same report found a biphasic relationship between duration of diet exposure and hippocampal BBB permeability, with heightened permeability during the first week of the diet, followed by a return to control levels after two weeks before increasing again after 4 weeks. 

To summarize, the findings described above indicate that the hippocampal BBB is highly sensitive to disruption following consumption of WD and similar energy rich diets. This BBB disruption can occur (in mice) after very short periods of diet exposure, and it appears to be more pronounced in the hippocampus compared to other brain areas. Less clear is whether BBB breakdown with obesogenic diets is based on properties of these diets per se, the amount of diet that is consumed, the obesity these diets produce, or some combination of these factors.

#### 2.11.2. Undernutrition

Determining the effects of undernutrition on the brains of nonhuman animals is complicated by the variety of deficiency manipulations (e.g., protein vs. energy), level of stress exposure, and the time course of the deficiency [437,438]. This literature presents a hippocampo-centric view of undernutrition, as this system has been widely used as an experimental model [439,440,441]. This focus has been to the exclusion of other brain areas, which are also clearly impacted by undernutrition [442,443]. There is some evidence that once damage is sustained to the hippocampus—and to the cerebellum—it may be difficult to reverse, in contrast to other structures [443]. In humans, chronic undernutrition during childhood results in a broad range of cognitive impairments [444], but these effects do not seem to be specific for HDLM [174]. Rather, it appears that extended periods of undernutrition exert adverse effects on all brain systems and there is little that distinguishes the hippocampus as unique.

Undernutrition and the BBB: There has been little research on the effects of undernutrition on the BBB. One study examining the transport of amino acids across the BBB following perinatal nutrient restrictions in rats found no evidence of BBB dysfunction [445]. However, another study investigated the effects of undernutrition on hippocampal BBB. In addition to finding that consuming a high-fat diet weakened the hippocampal BBB in mice, de Aquino [429] also showed that a protein-deficient diet disrupted BBB function in the mouse hippocampus. On postnatal day 10, dams were placed on hypo-protein diet, and the offspring were maintained on the same diet from postnatal day 21 (weaning) to postnatal day 52 (sacrifice). The permeability of the hippocampal BBB significantly increased, and the expression of claudin-5 was significantly reduced for the mice given the hypo-protein diet, relative to controls fed standard chow. 

Obesity/Obesogenic Diets and Undernutrition—Conclusions: Although it is difficult to separate the effect of obesity from the diets that induce it, there is some evidence that the hippocampus is selectively impacted by obesity, but there are no data indicating that it is impacted first. Considering the effects of WD and other obesogenic diets, the literature indicates that the hippocampus is the first and major target of this diet, which may imply its early involvement in obesity. There are many findings from rodent models that link obesity/obesogenic diets to a breakdown of the hippocampal BBB that is more pronounced, and may occur earlier, compared to BBB disruption in other brain areas. In contrast to this specificity, undernutrition has broad adverse effects on many brain systems. However, there is some evidence that a protein-deficient diet has harmful effects on the hippocampal BBB, but the data are limited. 

## 3. Implications

Table 1 summarizes the findings of the first part of the manuscript, where 11 domains and 22 exemplars of insults to which the hippocampus may be vulnerable are listed. For each insult we have attempted to categorize the evidence into three levels (Yes, No, ?-delineated by different colors in the table) indicating our conclusion about whether the available evidence favors that the hippocampus (leftmost part of Table 1), and the hippocampal BBB (rightmost part of the table) are affected at all, and if they are affected first and/or most, relative to other brain areas. A “?” indicates that the data were either equivocal or insufficient to make a determination of “Yes” or “No” for a given insult. 

Across domains and exemplars, the data presented in Table 1 indicate that, while the hippocampus is almost universally affected, whether it is affected first and/or foremost varied considerably. Nonetheless, in nine out of the eleven domains surveyed, the hippocampus was judged as being affected either first and/or foremost for at least one exemplar, which is more consistent with selective vulnerability than not. Table 1 also shows that, compared to the hippocampal vulnerability per se, there is less evidence across domains and exemplars that the hippocampal BBB is affected by insults, with more of the available data being indeterminant or indicating that it was not affected first or more than the BBBs of other brain areas. In short, there are a lot of missing data, and this is especially true for the BBB.

We can use the results summarized in Table 1 to identify not only the types of insults to which the hippocampus is most vulnerable, but also to consider the likelihood that such vulnerability is related to weakening of the hippocampal BBB. For the hippocampus, Table 1 identifies nine types of insults (HSV, epilepsy, ELS, PTSD, aging, AD, ischemia/hypoxia, TBI, obesity/obesogenic diets) that meet a selective vulnerability criterion of being affected first and more prominently compared to other sites in the brain. Table 1 also identifies three of these insults (PTSD, AD, obesity/obesogenic diets) with damage that occurs first and is more pronounced in the hippocampal BBB compared to the BBBs of other areas. This correspondence between hippocampal and hippocampal BBB pathophysiology suggests that the vulnerability of the hippocampus to PTSD, AD, and obesity/obesogenic diets may be more closely related to the breakdown of the hippocampal BBB than the vulnerability of the hippocampus to other insults is. To cast a wider—albeit weaker—net, one can consider additional insults with effects on the hippocampal BBB that are either first *or* more pronounced relative to the BBBs in other areas, for which the available data are indeterminant for one of these classifications. Of the remaining six insults that impact the hippocampus first and foremost, four (HSV, aging, ischemia/hypoxia, and MDD) are classified as affecting the hippocampal BBB either first or indeterminant, or more prominently or indeterminant. This raises the possibility that with further investigations at least some of the indeterminant findings for the BBB of these five insults will be resolved to strengthen their link to hippocampal vulnerability. Indeed, the findings summarized in Table 1 suggest where investigations may be most profitable.

The vulnerability of the hippocampus to insults also involves other factors that can act independently or in combination with the disruption of the hippocampal BBB. Oxidative stress, glutamate excitotoxicity, and neuroinflammation have been observed throughout the brain as consequences of exposure to a variety of insults, including those to which the hippocampus is selectively vulnerable [446]. While each of these processes are involved with beneficial and often protective functions, they can become sources of neurodegeneration and functional deficits when dysregulated [447,448]. For example, peripheral inflammation involves the release of proinflammatory cytokines, such as TNF-α, IL-6, and IL-1β, which can enter the brain via transport mechanisms and can also increase the permeability of the BBB [449]. With the infiltration of cytokines, microglia—the brain’s resident immune cells—are activated and can release additional quantities of cytokines and other neurotoxic molecules for long periods of time. This chronic neuroinflammation can cause or contribute to synaptic impairment, neuronal death and the exacerbation disease pathology [450]. Compared to other brain regions, the microglia population in the hippocampal formation is dense and the expression of proinflammatory cytokines in the hippocampus following peripheral immune challenges has been reported to be higher and faster compared to other brain regions [451,452]. These factors could increase the relative sensitivity of the hippocampus to the harmful effects of neuroinflammation compared to other brain regions and could worsen the adverse proinflammatory consequences of many insults to which the hippocampus is exposed.

Along with immune system components, hippocampal physiology and function are also influenced by a large and diverse array of neuropeptides, neurotransmitters, metabolic signals, and neurotrophic factors. Over 60 ligands with receptors or binding sites in the hippocampus have been identified [453]. Of these, endocrine signals have received much attention. Disturbances in these signaling systems can interfere with hippocampal functioning [454] with access to these hormones modulated by the BBB [455]. For example, in addition to their involvement in the regulation of energy intake, many studies show that reduced the availability of, or sensitivity to leptin, insulin, glucagon-like peptide-1 (GLP-1), and ghrelin is associated with impaired HDLM (for reviews, see [18,456,457]. 

Glucocorticoids (GCs) are steroid hormones that can freely cross the BBB after they are released from the adrenal cortex in response to stress. The discovery of receptors for stress hormones in the hippocampus [458] was followed by numerous studies demonstrating the adverse effects of chronic GC exposure on the brain with the highest neurotoxicity observed in the hippocampus [459]. It has also been proposed that chronic exposure to GCs, at levels that are not high enough to be neurotoxic on their own, could nonetheless “endanger” the hippocampus by making it more vulnerable to concurrent insults [460]. Consistent with this view, early hippocampal cell culture studies showed that the toxicity of excitotoxins [461], hypoglycemia, and hypoxia [462] were exacerbated by exposure to high GC levels.

Finally, damage to the hippocampus produced by some insults may bypass the BBB entirely. Transneuronal degeneration that can produce deterioration in the form of neuronal shrinkage, loss of dendrites and synapses, changes in axonal myelination, and death of neurons that are removed from the initial site of the insult [463,464]. In some cases, instead of direct disruption of distributed neural connections, the connections may serve as conduits for disease transmission [465,466]. Furthermore, disease transmission may not be the only cause of dysfunction at sites remote to the insult. Damaged regions may transmit disordered information to connected regions resulting in functional impairments [467]. 

## 4. General Conclusions

The purpose of this paper was to examine whether the hippocampus is differentially vulnerable to insult and to provide an assessment of the extent to which this vulnerability might involve breakdown of the hippocampal BBB. Across the most prevalent examples from all biomedical domains that impact the brain, the hippocampus was generally found not only to be affected, but also affected to a greater extent and earlier than other brain regions. Our findings also indicate that hippocampal vulnerability to many of these insults is accompanied by a loss of BBB integrity in this region. For some of these insults, there was evidence that weakening of the hippocampal BBB occurred before and was more pronounced compared to the BBBs of other brain areas. These conclusions are limited, especially when considering the hippocampal BBB, by a lack of relevant data or by equivocal findings, with respect to the effects of some insults. In addition to the need to more rigorously test the notion of unique hippocampal vulnerability, we conclude that addressing the questions of how the protections afforded by the hippocampal BBB are compromised and how that weakening impairs hippocampal functioning are research goals of major significance, given the wide range of insults to which the hippocampus is vulnerable.

## Figures and Tables

**Table 1:** Domains and exemplars of potential hippocampal and hippocampal BBB vulnerabilities.

	Hippocampus			Hippocampal BBB		
Domains and Exemplars	Affected	First	Most	Affected	First	Most
**Neuroactive Pathogens**						
Rabies	Yes	No	No	Yes	?	?
HSV	Yes	Yes	Yes	Yes	?	?
**Neurotoxins**						
Alcohol	Yes	No	No	Yes	?	?
Arsenic	Yes	?	Yes	Yes	No	No
OC/OP	Yes	?	?	?	?	?
Lead (Pb)	Yes	?	No	Yes	?	?
**Neurological Conditions**						
Epilepsy	Yes	Yes	Yes	Yes	No	No
Migraine	No	No	No	?	No	No
**Trauma**						
Early Life Stress (ELS)	Yes	Yes	Yes	?	?	?
PTSD	Yes	Yes	Yes	Yes	Yes	Yes
**Aging**	Yes	Yes	Yes	Yes	?	?
**Neurodegenerative disease**						
Alzheimer’s Disease	Yes	Yes	Yes	Yes	Yes	Yes
Parkinsons Disease	No	No	No	No	No	No
**Acquired brain injury**						
Ischemia/hypoxia	Yes	Yes	Yes	Yes	Yes	?
TBI	Yes	Yes	Yes	Yes	?	?
**Mental health conditions**						
MDD	Yes	?	?	Yes	?	Yes
GAD	Yes	?	?	?	?	?
**Endocrine disorders**						
Diabetes	Yes	Yes	?	Yes	?	?
Hypothyroidism	Yes	?	?	?	?	?
**Developmental disabilities**						
Down syndrome	Yes	No	No	?	?	?
ASD	Yes	?	?	No	No	No
**Nutrition**						
Obesity/obesogenic diets	Yes	?	Yes	Yes	Yes	Yes
Undernutrition	Yes	No	No	?	?	?

“?” denotes that the findings are either equivocal or sufficient data are lacking. Bold font is used for major domains and headers in the Table. Exemplars in each domain are not in bold font indicating that they are subcategories of each domain. Yes (Red), No (Green) ? (yellow) in regular font are used for the different classifications.

## Data Availability

Not applicable.
